# Supplementary material for: Minimum infusion rate and adrenocortical function after continuous infusion of the novel etomidate analog ET-26-HCl in rats
Source: PeerJ. 2017 Sep 4;5:e3693. doi: 10.7717/peerj.3693 (PMC5588785; doi:10.7717/peerj.3693)
Supplement: Data S1 [file peerj-05-3693-s001.pdf]

1 Minimum Infusion Rate

Animal number	Weight(g)	Drug	Infusion rate mg/(kg.min)	Rate ml/h	Begin of infusion	Total Infusion time	End of infusion	Result
Y202	286	Etomidate	0.45	3.90	10:13:05	0:40:00	10:53:05	+
I403	249	Etomidate	0.41	3.00	12:15:15	0:40:00	12:55:15	+
Y206	285	Etomidate	0.36	3.13	13:05:50	0:40:00	13:45:50	+
I405	238	Etomidate	0.33	2.30	14:21:30	0:40:00	15:01:30	+
Y208	290	Etomidate	0.30	2.60	15:13:36	0:40:00	15:53:36	+
I407	227	Etomidate	0.27	1.80	9:41:28	0:40:00	10:21:28	-
Y209	293	Etomidate	0.30	2.60	10:32:00	0:40:00	11:12:00	+
I409	230	Etomidate	0.27	1.90	12:33:01	0:40:00	13:13:01	-
Y207	269	Etomidate	0.30	2.40	14:37:21	0:40:00	15:17:21	+
J405	249	Etomidate	0.27	2.00	10:54:34	0:40:00	11:34:34	-
Z604	276	Etomidate	0.30	2.50	11:48:25	0:40:00	12:28:25	+
J407	234	Etomidate	0.27	1.90	13:34:30	0:40:00	14:14:30	-
Z602	277	Etomidate	0.30	2.50	14:25:52	0:40:00	15:05:52	+
J408	239	Etomidate	0.27	1.90	15:22:17	0:40:00	16:02:17	-
N409	273	ET-26-HCl	0.65	1.8	8:56:34	0:40:00	9:36:34	+
N410	263	ET-26-HCl	0.59	1.6	9:45:38	0:40:00	10:25:38	-
N401	280	ET-26-HCl	0.65	1.8	13:15:16	0:40:00	13:55:16	+
N405	266	ET-26-HCl	0.59	1.6	14:01:09	0:40:00	14:41:09	-
N404	266	ET-26-HCl	0.65	1.7	14:46:36	0:40:00	15:26:36	-
N407	280	ET-26-HCl	0.72	2	15:33:50	0:40:00	16:13:50	+
N402	285	ET-26-HCl	0.65	1.9	8:37:18	0:40:00	9:17:18	+
N408	292	ET-26-HCl	0.59	1.7	9:26:40	0:40:00	10:06:40	-
N403	264	ET-26-HCl	0.65	1.7	10:10:30	0:40:00	10:50:30	+
N406	279	ET-26-HCl	0.59	1.6	13:00:58	0:40:00	13:40:58	-
P902	229	ET-26-HCl	0.65	1.5	13:45:24	0:40:00	14:25:24	+
P905	229	ET-26-HCl	0.59	1.4	14:35:05	0:40:00	15:15:05	-
P708	349	CPMM	1.00	2.6	13:16:00	0:40:00	13:56:00	+
L50	254	CPMM	0.90	1.7	14:13:41	0:40:00	14:53:41	-
P702	323	CPMM	1.00	2.4	15:30:44	0:40:00	16:10:44	+
L502	247	CPMM	0.90	1.7	12:40:30	0:40:00	13:20:30	-
P709	320	CPMM	1.00	2.4	13:21:06	0:40:00	14:01:06	+
L503	263	CPMM	0.90	1.8	14:06:10	0:40:00	14:46:10	-
P707	322	CPMM	1.00	2.4	14:50:50	0:40:00	15:30:50	+
L510	257	CPMM	0.90	1.7	16:23:16	0:40:00	17:03:16	-
P706	342	CPMM	1.00	2.6	17:05:30	0:40:00	17:45:30	+
L508	264	CPMM	0.90	1.7	17:56:21	0:40:00	18:36:21	-

2 The level of serum corticosterone concentration

Etomidate

Time	μ003	μ001	μ002	μ008	G208	G208	G002	G104
0min	205.20	206.13	234.39	312.24	125.85	184.68	177.42	216.96
60min	37.77	35.04	105.66	64.71	18.57	18.42	28.05	27.66
90min	51.39	53.01	50.85	66.93	77.43	44.73	54.54	59.79
120min	64.38	62.49	56.13	47.73	112.68	53.46	81.09	91.95
150min	76.26	85.11	73.38	119.82	160.08	87.18	104.34	103.71
180min	83.40	112.26	67.56	108.87	158.07	111.36	79.29	131.85
210min	98.67	129.06	78.78	117.99	215.91	112.08	152.88	135.30
240min	123.72	146.07	84.81	208.65	237.93	98.79	196.89	118.26

ET-26-HCl

Time	T709	T706	μ006	μ004	G105	G207	G206	G101
0min	176.74	180.74	145.80	168.09	107.34	148.02	208.29	230.76
60min	43.08	52.89	101.37	88.05	116.91	70.83	278.13	194.70
90min	126.01	103.97	113.67	96.27	128.64	117.69	239.31	223.35
120min	210.28	149.58	151.05	170.70	184.53	141.18	269.34	333.15
150min	588.57	203.39	244.26	198.81	154.62	172.68	148.38	300.21
180min	300.09	192.24	230.70	204.96	222.54	251.40	169.47	500.67
210min	267.23	171.63	187.08	185.10	242.67	291.33	245.64	347.31
240min	244.74	310.30	294.72	203.10	247.83	249.66	278.28	487.86

CPMM

Time	μ007	T702	G202	V101	V105	G210	G203	G209
0min	196.65	381.81	129.18	90.90	74.73	187.08	121.5	133.32
60min	148.92	74.54	270.09	127.35	70.80	168.51	183.18	171.96
90min	378.09	290.45	222.45	189.63	147.36	270.24	232.5	272.52
120min	329.13	189.24	276.78	185.79	147.48	277.47	236.58	310.2
150min	327.72	577.99	210.39	168.36	148.05	640.20	299.52	261.3
180min	420.15	278.42	363.54	189.12	139.20	371.64	229.44	264.27
210min	402.18	332.66	348.09	293.19	192.72	243.93	234	270.6
240min	276.03	284.32	339.90	158.37	151.80	304.53	129.9	232.5

Control

Time	G008	G103	G203	Z604	Z603	Z605	G302	G303
0min	114.6	132.24	213.42	195.06	105.72	132.66	178.98	159.24
60min	176.58	231.48	272.52	0	164.46	244.62	227.46	186.36
90min	187.14	239.22	263.7	96.12	149.76	170.7	313.32	287.64
120min	283.8	311.76	390.54	141.9	144.9	170.7	437.88	329.52

150min	216.6	491.34	397.32	122.28	129.42	203.1	363.18	253.32
180min	431.34	154.08	109.8	106.68	159.72	141.66	307.08	275.28
210min	188.22	269.94	360.24	132.9	130.68	178.26	306.3	286.32
240min	209.46	358.26	388.32	79.74	144.66	177.96	459.84	257.16
